# Influence of Selected Transition Metals on Hard Magnetic Properties of Dy-Fe-Nb-B Vacuum Suction Rods

**DOI:** 10.3390/ma18194508

**Published:** 2025-09-28

**Authors:** Grzegorz Ziółkowski, Artur Chrobak, Ondrej Zivotsky, Joanna Klimontko

**Affiliations:** 1 Institute of Materials Engineering, University of Silesia in Katowice, 75 Pułku Piechoty 1A, 41-500 Chorzów, Poland; grzegorz.ziolkowski@us.edu.pl; 2 Department of Physics, VŠB-Technical University of Ostrava, 17 Listopadu 15/2172, 708 33 Ostrava-Poruba, Czech Republic; ondrej.zivotsky@vsb.cz; 3 Institute of Physics, University of Silesia in Katowice, 75 Pułku Piechoty 1A, 41-500 Chorzów, Poland; joanna.klimontko@us.edu.pl

**Keywords:** hard magnetic materials, ultra-high coercivity, vacuum suction casting

## Abstract

This study investigates the structural and magnetic properties of ultra-high coercivity (Fe_80_B_14_Nb_6_)_0.88_Dy_0.12_ alloys, doped with 0.5–5 at.% of selected metallic additions: magnetic (Ni, Co) and non-magnetic (Pt, Cu) elements. Material characterization involved both structural and magnetic measurements. Alloys containing dopant concentrations up to 2 at.% exhibited similar phase compositions, with the Dy_2_Fe_14_B compound being dominant. Magnetic hysteresis loops revealed a superposition of two components: magnetically soft and hard phases. A significant change in magnetic properties was observed within the 0.5 to 1 at.% dopant concentration range. Notably, the addition of 0.5 at.% Ni increased the apparent anisotropy field from 5.2 T to 7.5 T. Furthermore, 0.5 at.% Pt led to an increase in the coercive field from 4.6 T to 5.5 T. These additions influenced crystallization, resulting in the formation of a more regular microstructure without submicrometric dendrite branches, when compared to the base alloy.

## 1. Introduction

Magnetic materials are crucially important in modern technologies, including electric and electronic devices such as electric motors, generators, and refrigerators, as well as sensors, actuators, and spintronics [1,2,3,4,5]. Progress in these fields requires hard magnets with unique and breakthrough properties. Given this, the RE-Fe-Nb-B (RE = Tb, Dy) alloys align well with the research trend, constituting a new type of materials with ultra-high magnetic coercivity [6,7,8,9,10,11]. For example, the bulk (Fe_80_B_14_Nb_6_)_0.88_Tb_0.12_ alloy shows about 9 T coercive field at room temperature, which is related to the Tb_2_Fe_14_B phase and a specific microstructure formed by chemical composition as well as preparation method [6]. In this case, the aforementioned alloy was prepared using vacuum suction casting under favorable technology conditions. Samples of the alloy were crystallized from liquid phase in a copper mold with a cooling rate about 1000 K/s. Moreover, the alloying addition of Nb slows down crystallization rate, allowing the formation of irregular dendrites with submicrometric branches. The (Fe_80_B_14_Nb_6_)_0.88_Tb_0.12_ alloy exhibits relatively low magnetic saturation due to antiferromagnetic coupling between Fe and Tb magnetic moments. Nevertheless, the observed ultra-high coercivity can offer advantages in magnetic composites as a source of magnetic anisotropy [12,13,14]. By a combination of hard and soft magnetic properties, it is possible to optimize final magnetic characteristics desired for different applications, and therefore, the described alloy group is of particular importance.

Similar properties are observed in the family of Dy-Fe-Nb-B alloys. Tb and Dy belong to the same group of heavy rare earth elements and play the same role in the formation of RE_2_Fe_14_B (RE = Tb, Dy) phases, but dysprosium is economically more advantageous [15].

As previously mentioned, alloying additions are of crucial importance for the resulting microstructure and, as a consequence, hard magnetic properties. Recently, we have studied the influence of Zr on the crystallization and magnetic properties of (Fe_80_Nb_6_B_14_)_0.88_Dy_0.12_ alloys prepared by means of the vacuum suction casting technique [15]. It turned out that Zr as an alloying addition causes significant changes in final microstructure as well as phase composition. A high content of Zr (over 5 at.%) leads to the appearance of Tb-Fe and pure Fe separations coexisting with the hard magnetic Tb_2_Fe_14_B phase. This means that the alloy can be considered a composite, containing hard and soft magnetic components. Interactions between these phases are responsible for gradual changes in the magnetic characteristics observed with increasing Zr concentration, i.e., the maximum of energy product |*BH*|_max_, an increase in magnetic saturation, and a decrease in coercive field. The influence of low Zr content, especially up to 2 at.%, was particularly interesting. In this range, crystallization occurs in different ways which lead to the formation of different microstructures.

This study investigates the influence of low concentrations of selected transition metal additions (magnetic Ni and Co and nonmagnetic Cu and Pt) on the crystallization process and the resulting magnetic properties of Dy-Fe-Nb-B vacuum suction rods. The alloying additions were selected accounting for the possible formation of soft (FeNi, FeCo) or hard (FePt) magnetic phases as well as high thermal conductivity (Pt, Cu), which may influence the crystallization process in the technology used. The focus is on how these additions affect the hard magnetic phases, as revealed through magnetic characterization.

## 2. Materials and Methods

The base alloy of (Fe_80_Nb_6_B_14_)_0.88_Dy_0.12_ (denoted as Dy 12) was obtained using the arc-melting method by means of commercially available basic elements (purity of 99.9%). Furthermore, the samples were crushed and mixed with a proper composition of M = Ni, Co, Cu, Pt alloying additions, following the formula [(Fe_80_Nb_6_B_14_)_0.88_Dy_0.12_]_1−x_ M_x_ (*x* = 0; 0.005; 0.01; 0.015; 0.02; 0.03; 0.04; 0.05). Such specimens were melted and crushed several times in order to obtain the homogeneity in the melt. The next step consisted of the application of the vacuum mold suction technique. The technology procedure, carried out in a chamber with argon atmosphere, consists of the following steps: (i) the preliminary melted spherical piece of alloy was placed on top of the cooper mold and (ii) the sample was melted by electric arc (40 A) for 2 s and then (iii) rapidly sucked by vacuum into a hole (diameter of 1.5 mm) drilled in the mold. A detailed description of the technology used can be found in [16]. Finally, the samples took the form of rods 1.5 mm in diameter and 3 cm in length. All studied alloys and their abbreviations are listed in Table 1.

The structural properties of the samples were determined using the X-ray diffraction (XRD) technique. The XRD measurements were carried out utilizing a high-resolution Panalytical Empyrean diffractometer (Malvern Panalytical, Malvern, UK) with CuKα radiation (40 kV, 30 mA) equipped with a PIXcel detector (Malvern Panalytical, Malvern, UK). The data analysis was carried out using HighScore Plus software supplied by PANalytical (Version 4.9, Malvern Panalytical, Malvern, UK). The ICDD PDF-4 database was used to identify the phase composition.

The microstructure of was studied by means of a scanning electron microscope (SEM) JEOL JSM7600F (JEOL Ltd., Tokio, Japan) with an X-ray micro-probe. Pictures were collected using the back-scatter electron mode (BSE).

The magnetic characterization was carried out with the use of a SQUID magnetometer (XL-7, Quantum Design, Quantum Desing, San Diego, CA, USA) and MPMS magnetometer with the VSM option (Quantum Design, Quantum Desing, San Diego, CA, USA).

Magnetic domains were observed using the Nio-surf AFM (Nanosurf AG, Liestal, Switzerland) microscope with the MFM option (co-coated cantilever, type of Multi75Magnectic).

## 3. Results

**Figure 1:**
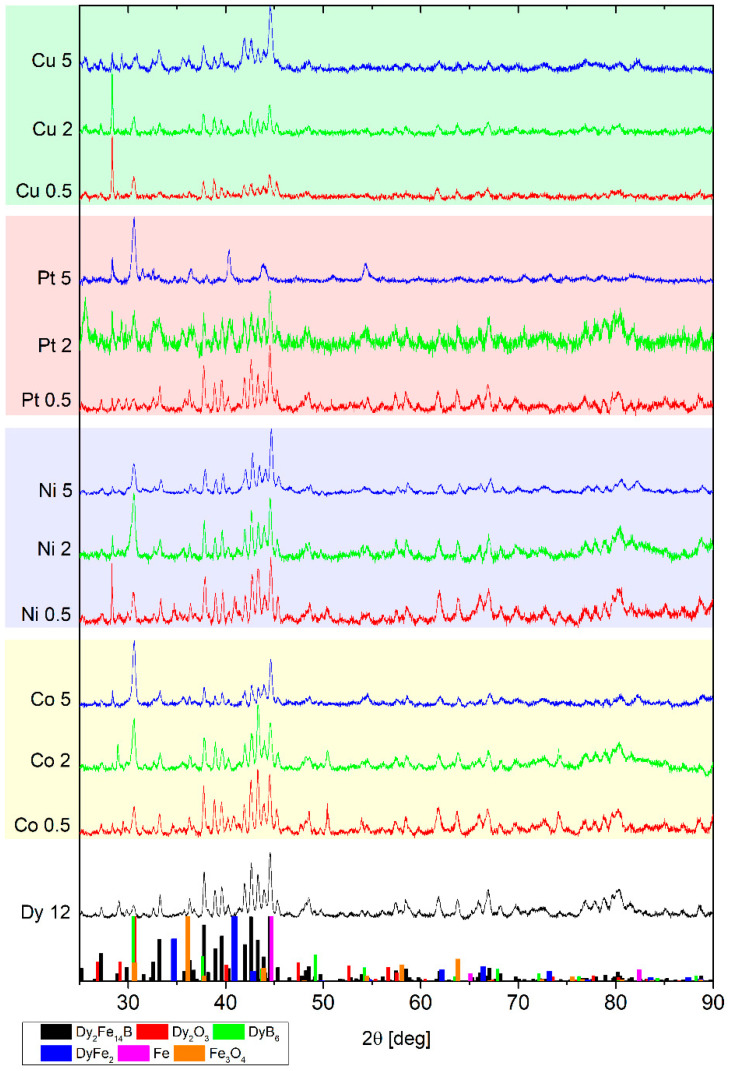
XRD patterns for all studied alloys with selected concentrations of the Ni, Co, Pt, and Cu additions. *Y*-axis expresses normalized number of counts (arbitrary unit). PDF card numbers: Dy_2_Fe_14_B—04-006-3170, Dy_2_O_3_—04-001-8748, DyB_6_—04-001-3711, DyFe_2_—04-019-7778, Fe—04-004-6370, Fe3O4—04-014-9664.

**Figure 2:**
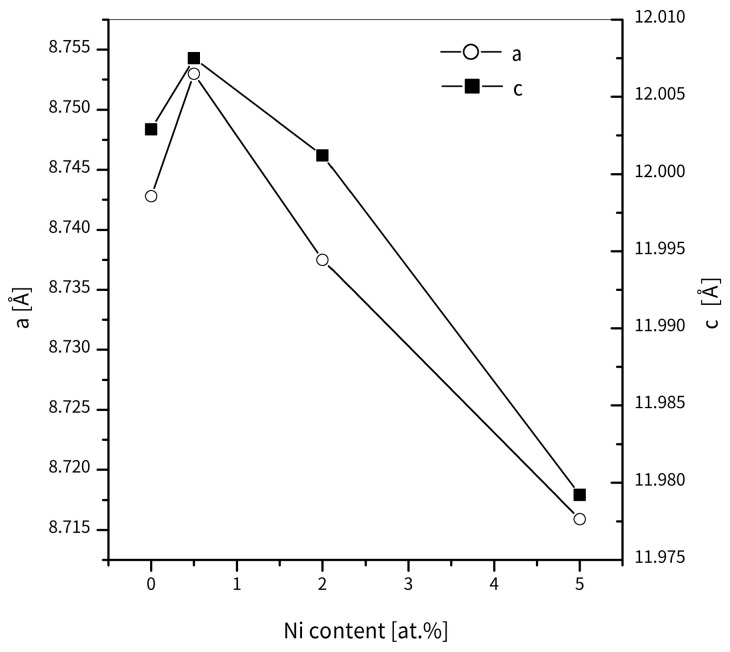
Lattice constants for Dy 12, Ni 0.5, Ni 2, and Ni 5 alloys determined by Rietveld refinement.

Figure 1 shows the XRD patterns obtained for all the types of alloys studied for selected (0.5, 2, and 5 at.%) concentrations of metallic additions. Additionally, the pattern for the basic alloy of (Fe_80_Nb_6_B_14_)_0.88_Dy_0.12_ is also included. Generally, the patterns consist of reflexes attributed to various phases of Dy_2_Fe_14_B, DyFe_2_, Fe, DyB_6_, and oxides (likely formed during sample preparation for the XRD measurements). One can also observe unassignable reflexes, which preclude a detailed determination of the complete phase composition. Nevertheless, a qualitative analysis is possible. For a low amount of the metallic additions, Dy_2_Fe_14_B was identified as the dominant compound. Notably, up to 2 at.% of the alloying additions, the phase composition remains similar across all examined cases. At 5 at.% Ni, Co, and Cu additions, the appearance of Fe and DyB_6_ reflexes was observed alongside the existing Dy_2_Fe_14_B phase. Contrary to this, 5 at.% of Pt caused the disappearance of the hard magnetic Dy_2_Fe_14_B phase as well as the formation of DyFe_2_ and DyB_6_. regarding hard magnetic properties, the Dy_2_Fe_14_B phase played the primary role, and for this reason, Rietveld refinement of the XRD patterns was carried out. The analysis focused on a lattice constant change in the function of the content of the alloying addition. Figure 2 presents such an analysis for the alloys group, with Ni as a representative case. The changes in the *a* and *c* axes are rather low, with a maxima of 0.5 at.% for Ni. It should be noted that the change in the *a* parameter is about one order of magnitude greater than the *c* parameter.

**Figure 3:**
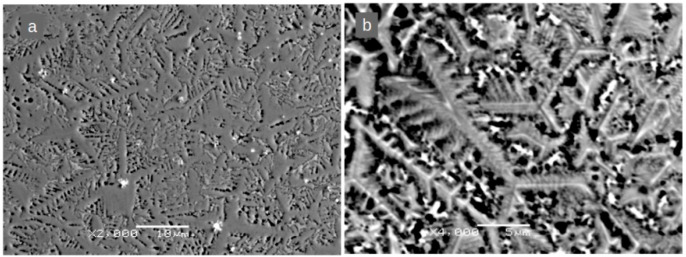
SEM BSE images for the (Fe_80_Nb_6_B_14_)_0.88_Dy_0.12_ alloy before (**a**) and after (**b**) NITAL treatment. (**a**) Magnification: ×2000, scale bar: 10 μm. (**b**) Magnification: ×4000, scale bar: 5 μm.

The microstructure of the base (Fe_80_Nb_6_B_14_)_0.88_Dy_0.12_ alloy is depicted in Figure 3a,b, which show scanning electron microscopy (SEM) images acquired in backscattered electron (BSE) mode (chemical contrast). The relatively high cooling rate during solidification resulted in the formation of irregular micrometric dendrites with submicrometric branches. The crystallization directions are more clearly discernible in Figure 3b, which displays the same sample after undergoing etching for a short time in a 2% NITAL solution (an etching solution made from nitric acid dissolved in ethanol). These dendrites exhibit an asymmetric star configuration, with planar angles of 120° between their cores and branches.

**Figure 4:**
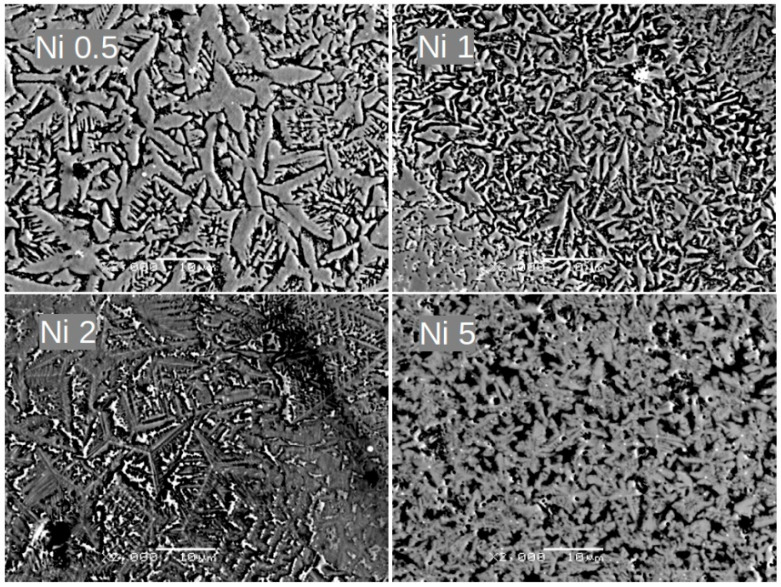
SEM BSE images for Ni 0.5, Ni 1, Ni 2, and Ni 5 alloys. Magnification: ×2000, scale bar: 10 μm.

**Figure 5:**
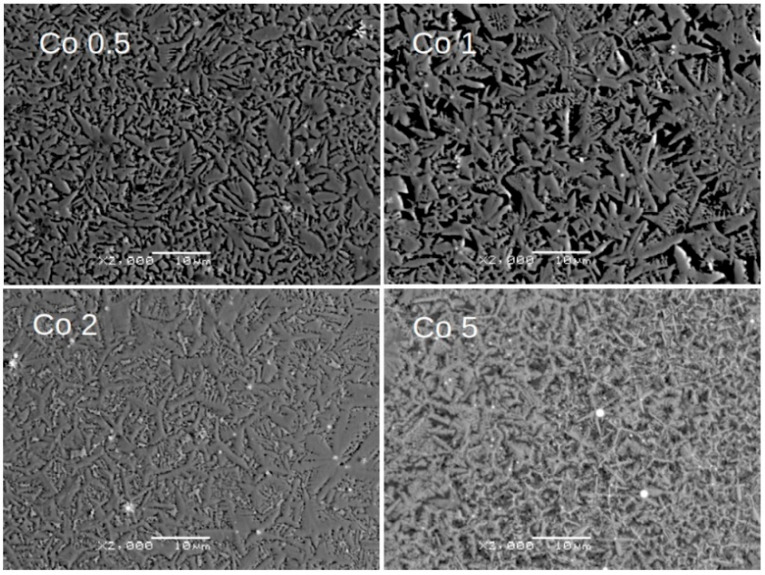
SEM BSE images for Co 0.5, Co 1, Co 2, and Co 5 alloys. Magnification: ×2000, scale bar: 10 μm.

**Figure 6:**
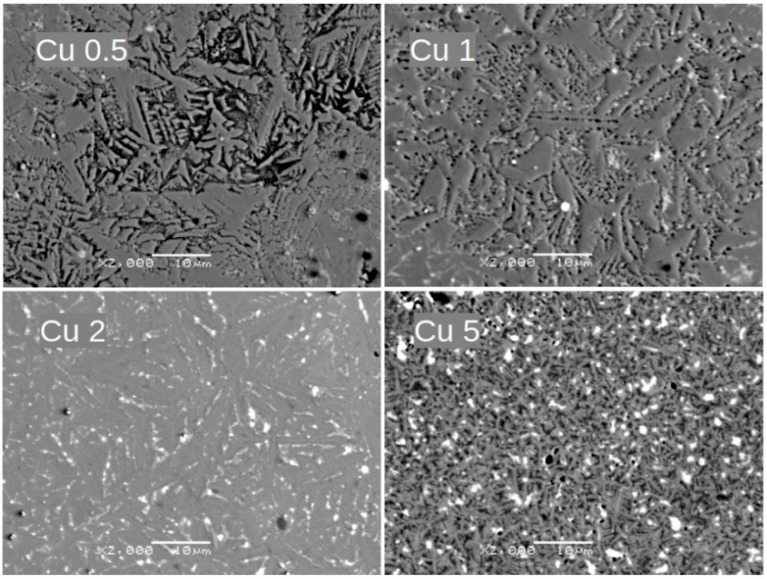
SEM BSE images for Cu 0.5, Cu 1, Cu 2, and Cu 5 alloys. Magnification: ×2000, scale bar: 10 μm.

**Figure 7:**
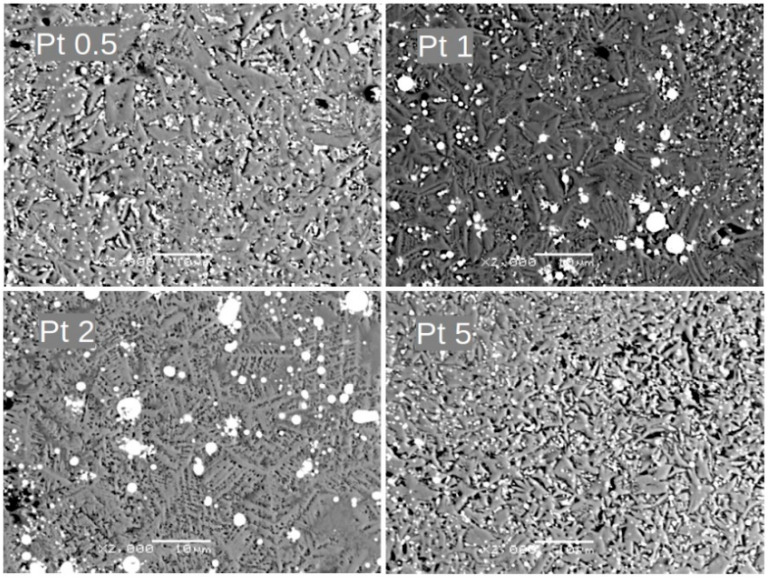
SEM BSE images for Pt 0.5, Pt 1, Pt 2, and Pt 5 alloys. Magnification: ×2000, scale bar: 10 μm.

Alloying additions of Ni, Co, Cu, and Pt significantly affect the alloy’s microstructure, as presented in Figure 4, Figure 5, Figure 6 and Figure 7, respectively. For samples with Ni, even a low concentration of 0.5 at.% leads to a remarkable change in grain morphology compared to the base alloy. The microstructure formed is notably more regular, and the submicrometric dendrite branches observed in Figure 2 have almost disappeared. Up to 2 at.% Ni, we observe a decrease in grain size. For higher Ni contents (i.e., 5 at.%), the microstructure is completely different, indicating a change in the crystallization pathway. Similar microstructural changes were observed with Co and Cu additions. However, in the case of 5 at.% Pt, where the XRD pattern revealed a significant change in the phase composition, the growing grains are less fragmented than with the other additions.

**Figure 8:**
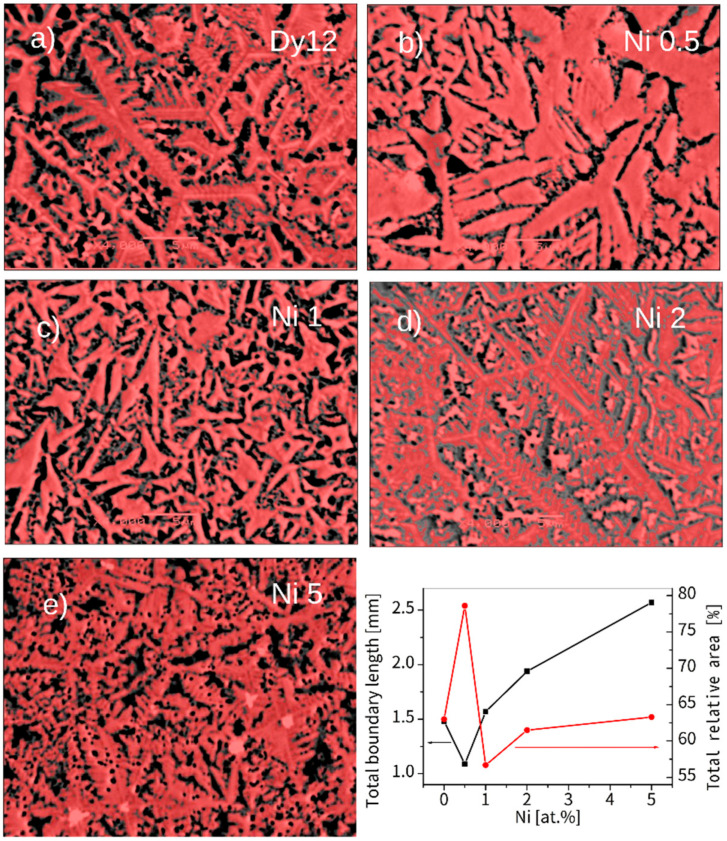
SEM BSE images for (**a**) Dy 12, (**b**) Ni 0.5, (**c**) Ni 1, (**d**) Ni 2, and (**e**) Ni 5 alloys after NIATL treatment. The red color indicates detected grains. The graph presents the total boundary length and relative area of the grains, determined from the SEM images. Magnification: ×4000, scale bar: 5 μm.

Interesting results can be obtained by the determination of the total boundary length and relative area of the detected grains based on the obtained SEM pictures for the samples after NITAL treatment (in order to emphasize grains boundary). These parameters effectively reflect an influence of the alloying additions on the development of grains. These quantities can be considered as densities because the analysis covered the same area. Figure 8a–e show the detected grains (colored in red) as well as the adequate dependencies of the aforementioned parameters for the alloys with Ni. As shown, the sequence of the grains’ development is confirmed. In fact, the total boundary length is at its minimum at 0.5 at.% and then gradually increases. This indicates a more regular shape and disappearance of the dendrite branches. The second parameter considered also revealed a minimum area between the grains for the sample with 0.5 at.% of Ni, which suggests their highest density and nearness.

**Figure 9:**
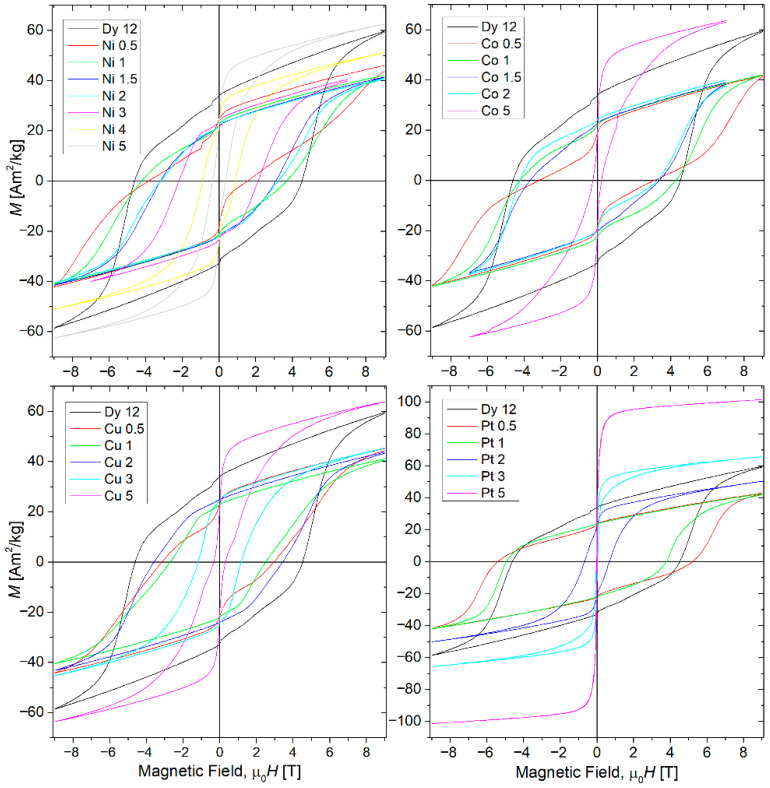
Magnetic hysteresis loops (determined at room temperature) for all studied cases.

The magnetic properties of the examined alloys were determined by measuring magnetic hysteresis loops at room temperature within an external magnetic field range of ±9 T. Additionally, thermomagnetic curves (from 10 K to 1200 K) were measured to identify magnetic transitions and illustrate the magnetic characteristics corresponding to phase composition changes. Figure 9 depicts the hysteresis loops for all studied cases. The curve for the base (Fe_80_Nb_6_B_14_)_0.88_Dy_0.12_ alloy has been included in each figure for comparison. All alloys exhibit hard magnetic properties with high and ultra-high coercivity. Notably, for low concentrations of the alloying additions, the hysteresis loops are asymmetric, meaning that the coercive field values differ in the negative (second quadrant) and positive (fourth quadrant) external magnetic fields. The loop shapes present one or two steps, indicating the presence of two magnetic components with distinct low and high anisotropy fields.

It is important to note that in all cases, with the exception of the Pt 5 sample, the hysteresis loops did not reach saturation even at an external magnetic field of 9 T. Furthermore, for samples with additions up to 2 at.%, there is no common magnetization trace; specifically, the magnetization curves diverge immediately after the reversal of the magnetic field direction. It is also interesting that the hysteresis loop of the Ni 0.5 sample is unclosed and highly asymmetrical.

**Figure 10:**
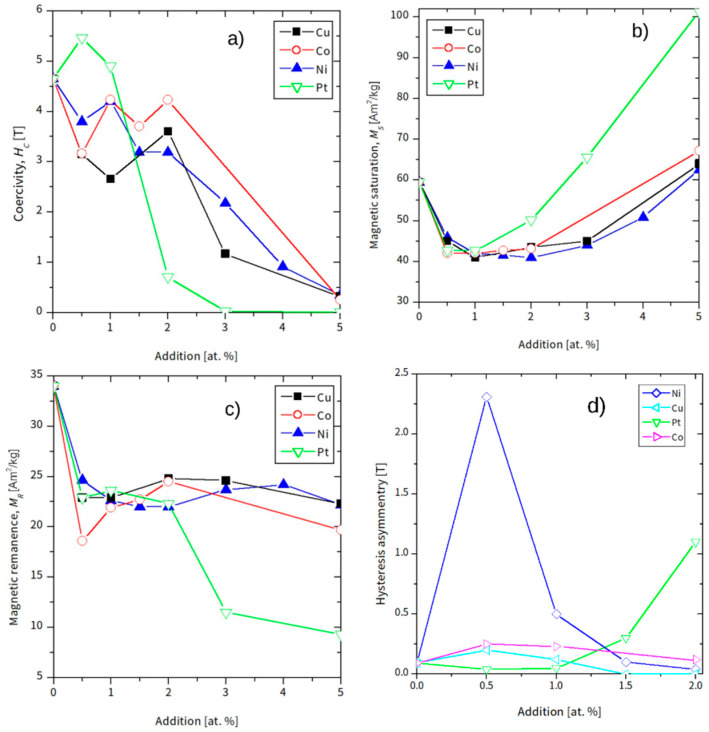
Coercive field Hc (**a**), magnetic saturation Ms (**b**), magnetic remanence MR (**c**), and asymmetry of coercivity (**d**), determined from hysteresis loops.

Selected magnetic parameters, including magnetic saturation (*M*_s_), magnetic remanence (*M*_R_), coercive field (*H*_c_) (determined in the second quadrant after the initial reverse magnetization process), and coercivity asymmetry (the difference between *H*_c_ in the second and fourth quadrants), are plotted in Figure 10.

A brief review of the hysteresis loops indicates that the magnetization processes of the studied alloys are complex. From a magnetic perspective, the magnetization results from the superposition and interactions between magnetic entities possessing different magnetic anisotropies, encompassing those with low, high, and ultra-high coercivity. The parameters presented in Figure 10 are not entirely independent because the samples could not be fully saturated. Consequently, analyzing these parameter values might introduce uncertainties or suggest physically unrealistic interpretations. Nevertheless, certain trends can be identified. As shown, the coercive field (*H*_c_) gradually decreases from approximately 4.6 T to 0.4 T with increasing alloying addition. Samples with platinum are exceptions, for which Hc initially increases to about 5.5 T (Pt 0.5 sample) and then sharply drops to nearly zero for Pt 3 alloys. Magnetic saturation (*M*_s_), defined in this analysis as the magnetization at the highest applied magnetic field, decreases up to 1 at.% of the alloying addition and subsequently increases gradually. The magnetic remanence (MR) decreases for low addition content and, for higher concentrations, remains at a relatively constant level, except for the alloys containing Pt. The observed asymmetry in the measured hysteresis loops is crucial for further analysis and discussion. The behavior of the Ni 0.5 sample is particularly unexpected, as a significant coercivity asymmetry (approximately 2.3 T) was detected. A similar effect was observed for (Fe_80_Nb_6_B_14_)_0.88_Tb_0.12_ and (Fe_80_Nb_6_B_14_)_0.88_Dy_0.12_ alloys obtained by vacuum suction technique [6,8]. These alloys belong to the magnetic materials with ultra-high coercivity. In the vicinity of high magnetic anisotropy and with an insufficient magnetic field applied to reach saturation, parts of magnetic moments can be stacked during the first pass of magnetization. For next passes of the hysteresis loop, these moments (or magnetic domains) are a source of exchange anisotropy and have influence on the magnetization processes (domain wall motion, pinning), resulting in the observed asymmetry of the hysteresis loops.

**Figure 11:**
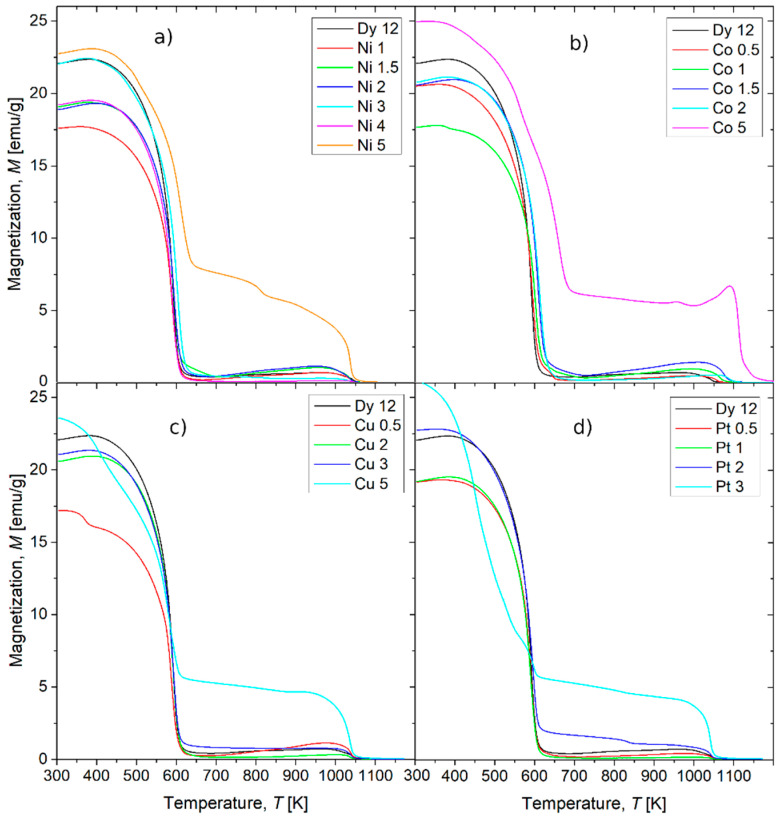
Thermomagnetic curves for the alloys with (**a**) Ni, (**b**) Co, (**c**) Cu, and (**d**) Pt alloying additions. The M(T) dependence for initial (Fe_80_Nb_6_B_14_)_0.88_Dy_0.12_ (denoted Dy 12) alloys as a reference is included to all graphs.

Figure 11 shows the thermomagnetic curves measured for all studied cases in the temperature range from 300 K to 1200 K. For low concentrations of the metallic additions, one can see a common behavior, i.e., the ferri- and para-magnetic transitions at about 600 K (typical for Dy_2_Fe_14_B) and gradual increase in magnetization up to the next Curie points at temperatures higher than 1050 K. These Curie temperatures suggest a thermally activated change in phase compositions leading to a separation of iron or its simple compounds. In the case of 5 at.% additions, the separation occurs in the alloys just after casting. From this part of the research, it can be concluded that the Dy_2_Fe_14_B phase is dominant in the samples with low amounts of additions and that its Curie temperatures are either independent or slightly increase with an increasing atomic contribution of the examined alloying additions.

**Figure 12:**
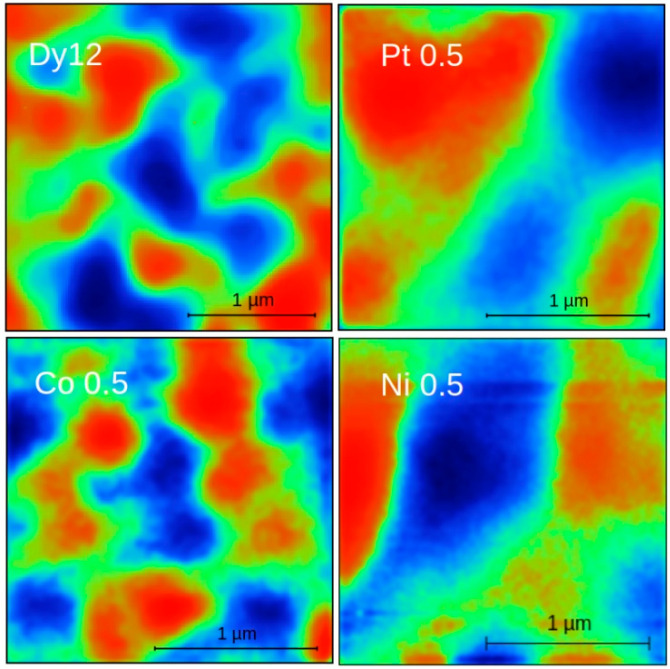
MFM pictures obtained for Dy12 (base alloy), Pt 0.5, Ni 0.5, and Co 0.5 alloys. Red and blue colors indicate attractive and repulsive force, respectively.

The magnetic domain structure was observed using the Magnetic Force Microscopy (MFM) technique. Given the unexpected magnetic effects observed in samples with 0.5 at.% additions, MFM images (Figure 12) are presented specifically for these alloys. Blue and red colors indicate repulsive and attractive forces, respectively, exerted on the magnetized AFM tip. The domains are irregular and range from micrometric to submicrometric in size. It is worth noting that for the Pt 0.5 and Ni 0.5 samples, the domains appear to be larger than those in the base alloy.

## 4. Discussion

Generally, RE_2_Fe_14_B phases are known as magnetic materials exhibiting hard magnetic properties. From an application point of view, the best known is Nd_2_Fe_14_B due to its relatively high remanence (about 120 emu/g), a coercivity less than 1 T, and an energy product |*BH*|_max_ of about 500 kJ/m^3^. This set of parameters is caused by its tetragonal crystal structure and ferromagnetic coupling between Nd and Fe magnetic moments. For Nd_2_Fe_14_B-doped systems, so-called heavy rare earth (HRE) elements (over gadolinium in the periodic table, e.g., Dy, Tb) lead to an increase in coercivity and simultaneous decrease in remanence as a consequence of strong spin–orbit coupling and antiferromagnetic alignment of RE-Fe magnetic moments, respectively. The RE_2_Fe_14_B phases with the HRE, supported by special technology and alloying additions, constitute another magnet group with ultra-high coercivity, exceeding 7 T at room temperature. Due to antiferromagnetic RE-Fe interactions, magnetic remanence cannot reach a high value, but this material family offers extreme resistance to reverse magnetization and can be used as a source of magnetic anisotropy in composite magnetic systems.

In this context, the vacuum suction-casting method utilized in this study is particularly interesting due to the relatively high cooling rate (approximately 10^3^ K/s) achieved during solidification from the liquid state [17,18,19]. These conditions, combined with an appropriate chemical composition, enable the synthesis of materials with specific phase compositions and microstructures, both typically responsible for unique properties. In this context, Fe, B, and Dy contribute to the formation of the hard magnetic Dy_2_Fe_14_B compound, while Nb slows down the crystallization rate, thereby preventing the enlargement of growing grains. The primary aim of incorporating metallic magnetic and nonmagnetic additions is to influence the phase composition and/or microstructure of the resultant alloy. The key points influencing the choice of magnetic additions (ferromagnetic Ni, Co) was the consideration of (i) the possible formation of soft magnetic phases or separations and (ii) slowing down the formation of RE_2_Fe_14_B for it to main in a hard magnetic phase. The non-magnetic elements were selected for a comparison with the magnetic additions. The following features are of particular importance; (i) Pt can form a PtFe hard magnetic compound, (ii) Pt and Cu have high thermal conductivity which can influence thermodynamic properties during casting, (iii) Pt and Cu do not substitute Fe in the RE_2_Fe_14_B phase and, therefore, they could be an obstacle to the fast crystallization process.

XRD measurements (Figure 1) indicated that the complete phase composition is not precisely known, owing to the presence of unidentified reflections and imperfect fitting to patterns of possible compounds. Nevertheless, Dy_2_Fe_14_B was detected as a dominant phase for alloys containing the additions below 5at.%. This observation is corroborated by the thermomagnetic curves, which are highly sensitive to the presence of different phases with distinct Curie temperatures. The M(T) dependencies (Figure 11) primarily show two Curie points: one at approximately 600 K, attributable to Dy_2_Fe_14_B, and a second one above 1050 K, corresponding to Fe. The employed alloying additions did not significantly affect these Curie temperatures, with the exception of alloys containing 5 at.% additions, where an increase in this parameter was observed. Generally, for x < 0.05, the phase composition remains similar, and therefore, changes in magnetic properties cannot be solely attributed to this factor.

Another important factor influencing magnetic properties is the microstructure. As shown for the base alloy (Figure 3), the microstructure consists of dendritic grains with well-developed branches. The crystallization front originates from a nucleation site and propagates in three directions, forming a star configuration, which is clearly visible in Figure 3b for the sample after undergoing etching for a short time in the NITAL solution.

Notably, a similar evolution of grain morphology is observed for alloys with the incorporated additions. Surprisingly, even a low concentration of 0.5 at.% addition significantly alters the shape of the growing grains. These grains are more regular and lack the submicrometric dendrite branches observed compared to the (Fe_80_Nb_6_B_14_)_0.88_Dy_0.12_ base alloy. This effect is particularly evident in samples containing Ni and Pt. Further increasing the concentration of the addition up to 2 at.% leads to a gradual fragmentation of the grains. Finally, 5 at.% of the additions results in a completely different microstructure. The grains become submicrometric (or even mesoscopic) and coexist with separated phases of the additions. It should be emphasized that this described microstructural evolution is similar across all studied cases.

The primary objective of this research was to investigate the magnetic properties of the alloys in question. The measured hysteresis loops served as the source for determining various magnetic parameters. A key finding is that most hysteresis loops remain unsaturated for the magnetic fields used (with the exception of samples containing 5 at.% of the addition) and, in some instances, are unclosed and asymmetric. This behavior is characteristic of hard magnetic materials with ultra-high coercivity. The challenge in precisely determining magnetic parameters arises from the fact that not all magnetic domains align with the applied field direction. For example, the magnetic saturation (*M*_s_), defined as the magnetization value at the highest applied magnetic field, decreases up to 2 at.% of the additions. While there is not a straightforward physical explanation for this effect, it can be understood by considering that not the entire sample volume reaches saturation. Paradoxically, the presence of certain magnetic entities with an anisotropy field exceeding the applied external magnetic field leads to a measured decrease in both saturation (Figure 10b) and remanence (Figure 10c).

**Figure 13:**
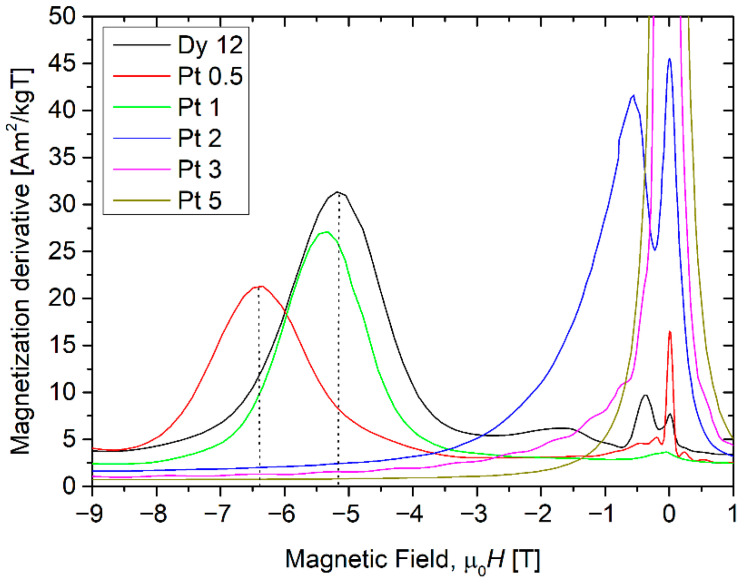
d*M*/d*H* curves for Pt alloying additions. The dependence for initial (Fe_80_Nb_6_B_14_)_0.88_Dy_0.12_ (denoted Dy 12) alloys is included in the graph as a reference.

Analysis of the hard magnetic objects and their changes with increasing alloying additions can be carried out based on d*M*/d*H* dependencies. Such quantity, calculated during so-called reverse magnetization process (hysteresis loop from +*H*_max_ to −*H*_max_), reveals that different magnetic objects that can be analyzed separately. Moreover, the shape of d*M*/d*H* reflects the distribution of the objects, and usually has a maximum/maxima. These maxima can be considered as apparent anisotropy field characteristics for the magnetic objects grouped into distributions. Figure 13 shows an example of the d*M*/d*H* curve for the alloys with Pt. The maxima placed near zero magnetic field are caused by magnetically soft objects while the maxima in the higher field depict hard magnetic objects formed during casting. The maxima for the exemplary alloy sample indicate the magnetic hardening caused by 0.5 at.% of Pt. The apparent anisotropy field increases from about 5.2 T to 6.4 T.

**Figure 14:**
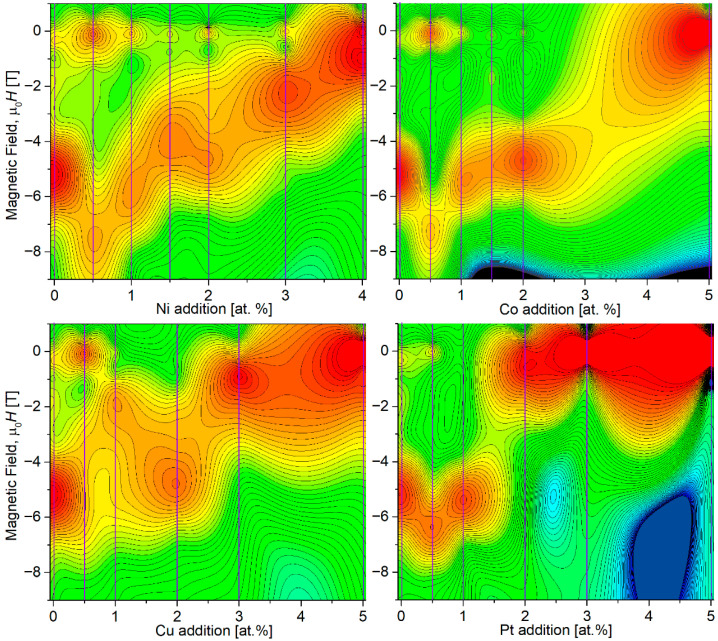
d*M*/d*H* (marked by color) in a function of the additions concentration and applied external magnetic field. Colors from blue to red indicate increasing values of d*M*/d*H*.

Based on this idea, one can plot d*M*/d*H* (marked by color) in a function of the additions concentration and applied external magnetic field (see Figure 14). This type of graph serves as a valuable tool for visualizing the magnetic properties of distinct phases with varying magnetic responses. It enables the determination of the number of these magnetic constituents and their specific properties, such as anisotropy field and distribution shape, that collectively contribute to the overall magnetic behavior of the sample.

**Figure 15:**
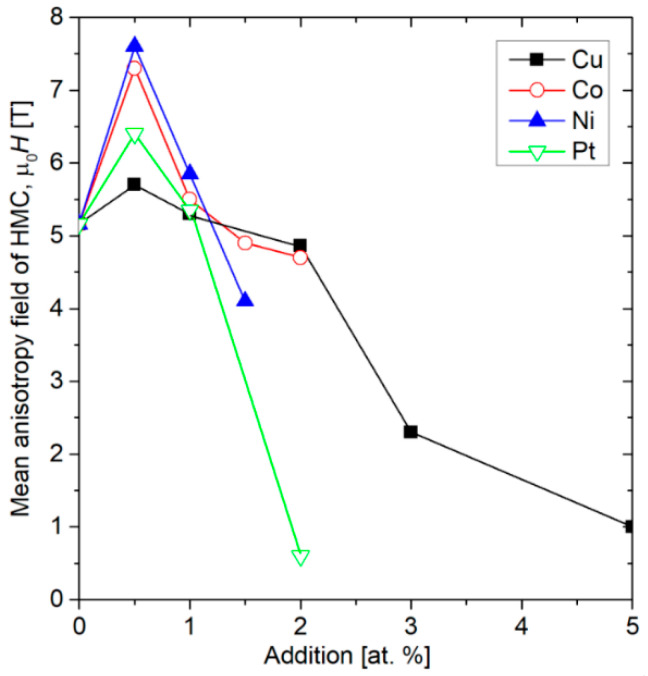
Mean anisotropy field of the hard magnetic component (HMC) for (Fe_80_Nb_6_B_14_)0_.88_Dy_0.12_ doped with Ni, Co, Cu, and Pt.

The alloys containing Ni and Pt exhibit particularly interesting characteristics. We observe two primary types of magnetic entities: a dominant hard component (initially centered around 5.2 T) and a soft component (around 0 T). The 0.5 at.% addition of Pt or Ni leads to significant hardening of the hard component. Specifically, the hard component’s coercivity increases from 5.2 T to 6.4 T for Pt and 7.5 T for Ni. Concurrently, an enhancement of the soft magnetic component is noted in the Ni 0.5 sample. This explains why the overall coercivity of the Ni alloys decreases. Conversely, in the Pt 0.5 sample, the soft component does not grow, resulting in an increase in the coercive field from 4.6 T to 5.5 T. For the second group of alloys, those with Co and Cu, the soft component shows a maximum at 0.5 at.% and then nearly disappears up to 3 at.% of the additions. This effect accounts for the initial decrease in coercivity followed by a subsequent increase, as illustrated in Figure 10a. At higher concentrations of these additions, the hard components lose their coercivity, leading to a magnetically softer response with coercive fields around 0.4 T. Figure 15 presents the values of the mean anisotropy field of the hard magnetic component for (Fe_80_Nb_6_B_14_)_0.88_Dy_0.12_ doped with Ni, Co, Cu, and Pt, showing the quantitative influence of the used additions.

In conclusion, the influence of the addition of a small amount of alloying elements on the magnetic properties of the (Fe_80_Nb_6_B_14_)_0.88_Dy_0.12_ alloy is a complex phenomenon. Notably, low concentrations of additions, up to 2 at.%, do not alter the phase composition, as confirmed by XRD and thermomagnetic measurements. However, these additions significantly change the magnetic properties, suggesting that the origin of this effect is linked to the specific microstructure formed during casting. The SEM observations indicate that the alloying additions influence the crystallization front, leading to the formation of more regular grains without submicrometric dendrite branches. This is particularly evident in samples with 0.5 at.% additions, especially for Ni (see Figure 4). It is well-established that hard magnetic properties are highly dependent on grain shape. A key factor is the ratio between magnetic moments located on the surface and within the volume of a grain. The direction of magnetic anisotropy at the surface differs from the direction of magnetic anisotropy in the volume (it can be placed parallel or perpendicular to the surface, depending on the sign of the surface anisotropy energy) [20]. This implies that a portion of magnetic moments on the surface can be reversed at a lower magnetic field compared to those within the grains’ volumes. Exchange interactions between the surface and volume lead to an averaging effect on the apparent magnetic anisotropy, which often results in the deterioration of hard magnetic properties. This scenario has been supported by both experimental and theoretical investigations [21,22,23,24]. In this context, the alteration of grain shape is responsible for the magnetic hardening of the hard magnetic component, as demonstrated in Figure 15. Conversely, higher concentrations of the additions induce observable phase composition changes (such as the separation of Fe in the Pt-containing sample), lead to the formation of a qualitatively different microstructure, and enhance soft magnetic properties.

## 5. Conclusions

In relation to the (Fe_80_Nb_6_B_14_)_0.88_Dy_0.12_ alloy, the conclusions can be summarized as follows:

- Phase Structure Stability: Small amounts of alloying additions (Ni, Pt, Co, Cu) do not significantly affect the phase composition, where the hard magnetic Dy_2_Fe_14_B phase remains the dominant component.
- Microstructural Sensitivity: The microstructure of the studied alloys is highly sensitive to these additions. It was demonstrated that 0.5 at.% additions alter the crystallization front, resulting in a microstructure containing hard magnetic grains with remarkably different shapes, specifically lacking submicrometric dendrite branches.
- Dual Magnetic Components: Magnetically, the investigated alloys are composed of two distinct magnetic components, one exhibiting soft magnetic properties and another that is magnetically hard with a coercivity exceeding 5 T.
- Microstructure-Induced Hardening: The observed regular shape of the hard magnetic grains (in samples with 0.5 at.% of the additions) is responsible for further magnetic hardening of the hard magnetic component. Notably, 0.5 at.% Ni proved most effective for enhancing the hard magnetic properties of this component, whereas 0.5 at.% Pt enhanced the hard magnetic properties of the entire alloy, with its coercivity increasing from 4.6 T to 5.5 T.
- Deterioration at Higher Concentrations: Higher concentrations of the tested additions (above approximately 2 at.%) lead to changes in both phase composition and microstructure, which subsequently result in the deterioration of hard magnetic properties.

## Figures and Tables

**Table 1:** List of alloys studied and their abbreviations used (the number means nominal concentration of the metal in at.%).

Abbreviation	Alloy
Ni 0.5, Ni 1, Ni 1.5, Ni 2,Ni 3, Ni 4, Ni 5	[(Fe_80_Nb_6_B_14_)_0.88_Dy_0.12_]_1−x_ Ni_x_
Co 0.5, Co 1, Co 1.5, Co 2,Co 3, Co 4, Co 5	[(Fe_80_Nb_6_B_14_)_0.88_Dy_0.12_]_1−x_ Co_x_
Cu 0.5, Cu 1, Cu 1.5, Cu 2,Cu 3, Cu 4, Cu 5	[(Fe_80_Nb_6_B_14_)_0.88_Dy_0.12_]_1−x_ Cu_x_
Pt 0.5, Pt 1, Pt 1.5, Pt 2,Pt 3, Pt 4, Pt 5	[(Fe_80_Nb_6_B_14_)_0.88_Dy_0.12_]_1−x_ Pt_x_

## Data Availability

The original contributions presented in this study are included in the article. Further inquiries can be directed to the corresponding author.
